# Acetylcholinesterase blocks cleavage of APP by γ-secretase in 293 cells and mouse brain

**DOI:** 10.1186/1750-1326-7-S1-S11

**Published:** 2012-02-07

**Authors:** Xin Niu, Xuejin Zhang, Jing Xie, Xuejun Zhang

**Affiliations:** 1 State Key Laboratory of Molecular Cell Biology, Institute of Biochemistry and Cell Biology, Shanghai Institutes for Biological Sciences, Chinese Academy of Sciences, Shanghai 200031, China

## Background

γ-Secretase cleaves amyloid precursor protein (APP) to produce Aβ peptide, and the secretase is a critical factor in the pathogenesis of Alzheimer’s Disease (AD), whereas acetylcholinesterase (AChE) is a key factor for maintaining normal nerve impulses. However, there has been no published research that focuses on the relationship between these two factors. γ-Secretase cleaves various substrates, and loss of function of γ-secretase is lethal. Thus, the mechanism by which γ-secretase cleaves APP is a key factor in AD pathogenesis. AChE is one of the main targets in the treatment of AD, and AChE inhibitors are commonly used for AD. Currently there is agreement that AChE inhibitors stabilizes acetylcholine (ACh) levels in the brain, which ensures ACh transport among neurons. However, the actual effect is limited. It has been reported that AChE interacts with PS1 and affects its glycosylation. This result suggests a link between AChE and γ-secretase. Here, we have tested the effects of AChE on cleavage of APP by γ-secretase.

## Method

We therefore investigated the function of γ-secretase in cleaving APP in AChE knockout mice (AChE+/−) and their wild-type (AChE+/+) counterpart. The γ-secretase assay was performed according to Farmery’s method published in 2003. In vivo data were checked with *in vitro* experiments, in which we overexpressed human AChE in 293 cells, and compared the function of γ-secretase of the cells with control. At last, in order to form the causal relation between AChE and γ-secretase, we use siRNA to knock down AChE to observe whether the cleavage of APP by γ-secretase could be rescue.

## Result

We observed stronger APP cleavage by γ-secretase in AChE knockout mice than in the wild-type counterpart, which suggests that AChE could block cleavage of APP by γ-secretase to some extent. This result is consistent with our in vitro data, which showed that cleavage of APP by γ-secretase was partially inhibited in 293 cells transfected with AChE, but that the inhibition could be rescued by siRNA-targeted AChE. These results indicate that there is a relationship between AChE and γ-secretase.

## Conclusion

Our data confirms that AChE functions in AD and we are the first to report a link between the APP-cleaving function of γ-secretase and AChE. Moreover, AChE is downregulated in the brains of AD patients, compared with healthy old people. This finding also suggests that AChE and γ-secretase might be inversely correlated.

